# Effect of formononetin on progressive pulmonary pathologies: multitarget mechanisms and therapeutic prospects

**DOI:** 10.3389/fphar.2025.1645964

**Published:** 2025-10-22

**Authors:** Dan Yan, Saibin Wang

**Affiliations:** Department of Pulmonary and Critical Care Medicine, Jinhua Municipal Central Hospital, Jinhua, Zhejiang, China

**Keywords:** *formononetin*, lung injury, COPD, asthma, pulmonary arterial hypertension, pulmonary fibrosis

## Abstract

*Formononetin* (FMN), an isoflavone derived from Radix Astragali and red clover, has promising therapeutic potential for a wide spectrum of respiratory diseases, including acute lung injury (ALI), pulmonary arterial hypertension (PAH), chronic obstructive pulmonary disease (COPD), asthma, and pulmonary fibrosis (PF). Mechanistically, FMN alleviates oxidative stress, inflammation, and fibrotic remodelling by activating Nrf2/HO-1, inhibiting NF-κB, and modulating the activity of the TGF-β/Smad signalling pathway. Evidence from cellular and animal studies has shown that FMN attenuates lung injury, prevents vascular remodelling, and slows the progression of fibrosis. However, its clinical translation is hampered by poor solubility, rapid metabolism, and low oral bioavailability, which limit its therapeutic effectiveness. To overcome these challenges, novel delivery systems—such as albumin-based FMN nanoparticles (FMN@BSA nanoparticles)—have been developed to increase the stability, bioavailability, and pharmacological potency of FMN. Despite encouraging preclinical outcomes, further studies are needed to clarify upstream mechanisms and conduct rigorous clinical evaluations. This review highlights the potential of FMN as a novel therapeutic candidate for respiratory diseases by summarizing its mechanisms of action and underscoring the importance of advanced delivery strategies in facilitating its future clinical application.

## 1 Introduction

Progressive pulmonary diseases, including ALI, asthma, COPD, PAH and PF, collectively account for substantial morbidity and mortality and remain the leading cause of disability-adjusted life-years lost worldwide (Wynn and Ramalingam, 2012; Pradhan et al., 2025; Sankari et al., 2025; Wang et al., 2022). Despite advances in pharmacotherapy, current treatments often fail to effectively relieve inflammation and fibrosis caused by endothelial barrier disruption, macrophage pyroptosis, and inflammatory response dysregulation, resulting in incomplete disease management (Cheng et al., 2021; Zhao et al., 2017). For instance, inhaled corticosteroids and bronchodilators manage symptoms in patients with asthma and COPD but do not stop disease progression or reverse structural changes. Similarly, antifibrotic treatments for PF, such as nintedanib and pirfenidone, slow progression but have limited efficacy and side effects. Furthermore, the pharmacological effects of conventional treatments are limited by problems such as poor drug delivery to injured lung tissues due to impaired microcirculation and elevated interstitial fluid pressure (Huang et al., 2020). These findings underscore the urgent need for new therapies that can effectively target multiple pathological pathways with improved pharmacological efficacy and safety.

*Formononetin* (FMN, 7-hydroxy-4′-methoxyisoflavone), a phytoestrogen in botanical drugs such as *Astragalus mongholicus* Bunge [Fabaceae; *Astragali radix*] (Radix Astragali) and *Trifolium pratense* L. [Fabaceae; *Trifolii pratensis flos*] (red clover), has recently attracted considerable attention as a promising multifunctional agent for the treatment of lung diseases. While FMN exerts broad pharmacological effects in various conditions (such as cancer, atherosclerosis, kidney injury) (Ma and Wang, 2022; Li et al., 2015; Lv et al., 2020; Gautam et al., 2017; Chen et al., 2025; Dong et al., 2022), this review focuses exclusively on its therapeutic potential for pulmonary pathologies, driven by its potent anti-inflammatory, antioxidant, and antifibrotic properties. For example, in mouse models of hyperoxia-induced ALI, oral FMN increases Nrf2 nuclear translocation, reduces neutrophil influx, and improves arterial oxygenation (Chen et al., 2021). In asthma models, it decreases goblet-cell hyperplasia, collagen-I deposition, and airway hyperresponsiveness by inhibiting NF-κB and JNK signalling (Yi et al., 2020). Preliminary studies on COPD, PAH, and PF suggested that FMN reduces TGF-β1, MMP-2/9, and NLRP3 inflammasome activity while increasing endothelial nitric oxide levels (Cai et al., 2019; Li et al., 2024; Ouyang et al., 2023). These findings highlight the potential of FMN as a versatile agent for the treatment of inflammation and fibrosis in lung diseases.

In addition to its inherent pharmacological properties, emerging evidence indicates that optimizing drug delivery strategies can significantly increase the therapeutic efficacy of FMN. FMN delivered by albumin-based nanoparticles (FMN@BSA nanoparticles) and inhalable FMN-loaded PLGA large porous microspheres (FMN-PLGA-MSs) have been developed to increase the accumulation of the drug in the lungs, reduce fibrosis, and sustain pulmonary release. These systems demonstrate that structural modifications and advanced nanocarriers can increase efficacy by overcoming the low solubility and oral bioavailability of FMN (Ouyang et al., 2023; Liu et al., 2025).

This mini-review aims to synthesize and critically analyse existing *in vitro* and *in vivo* evidence on the therapeutic potential of FMN across major pulmonary pathologies, highlight consistencies in the pathways involved, identify research gaps, and propose concrete directions for future research.

## 2 FMN protects against ALI by regulating oxidative stress, inflammation, and cell death pathways

ALI and its more severe manifestation acute respiratory distress syndrome (ARDS) represent critical conditions precipitated by a variety of insults, including infection, trauma, shock, and inhalation of toxic gases. The fundamental pathological processes involved are increased oxidative stress, dysregulated inflammatory responses, and multiple forms of programmed cell death, such as apoptosis, pyroptosis, and ferroptosis. These processes ultimately lead to disruption of the alveolar–capillary barrier and compromised gas exchange. Such severe damage can result in pulmonary oedema and, ultimately, respiratory failure. FMN, an isoflavone predominantly found in red clover and other legumes, has attracted considerable attention because of its protective effects against lung damage, as demonstrated in both *in vitro* and *in vivo* models.

FMN consistently exerts protective effects in various animal models of ALI by targeting the interconnected pathways of inflammation, oxidative stress, and cell death. Among antioxidant pathways, FMN effectively activates the Nrf2/HO-1 pathway, thereby mitigating hyperoxia-induced acute lung injury (HALI) in mice and pulmonary injury in a rat model of acute pulmonary embolism (Chen et al., 2021; Zhao et al., 2023). HO-1, an enzyme activated by Nrf2, is crucial for cellular defence because it decreases oxidative stress, regulates inflammation, and inhibits apoptosis via biliverdin, carbon monoxide, and free iron production (Constantin et al., 2012). Nrf2 functions as a significant transcription factor in oxidative stress and can affect the activation of antioxidant responses through a range of cytoprotective and antioxidant enzyme-encoding genes. Nrf2 activation has been shown to protect against various lung injuries by mitigating oxidative damage, abnormal inflammation, and apoptosis (Zhao et al., 2017). In terms anti-inflammatory pathways, FMN significantly decreases inflammatory cell infiltration in bronchoalveolar lavage fluid (BALF), increases superoxide dismutase (SOD) activity, and inhibits myeloperoxidase (MPO) activity in lipopolysaccharide (LPS)-induced mouse models of ALI (Ma et al., 2013). MPO is highly active in neutrophil granulocytes and produces hypohalous acids that mediate antimicrobial activity. Mechanistically, FMN alleviates septic ALI in rats by suppressing the HMGB1/RAGE/NF-κB signalling pathway, resulting in a reduction in the expression of key proinflammatory cytokines, including TNF-α, IL-1β, and IL-6 (Li, 2024). Specifically, FMN enhances the activity of SIRT1, which deacetylates NF-κB subunit p65 to inhibit its nuclear entry and reduces the release of HMGB1 from macrophages, thereby blocking the activation and amplification of NF-κB signalling. With respect to cell death modulation, FMN, particularly in nanoparticle formulations, strongly inhibits macrophage pyroptosis by directly blocking NLRP3 inflammasome activation (Ouyang et al., 2023). This leads to the downregulation of proinflammatory cytokines and the inactivation of fibroblasts, thereby attenuating immune responses and extracellular matrix (ECM) deposition. Furthermore, FMN inhibits LPS-induced apoptosis of alveolar epithelial cells and ameliorates inflammatory responses, a process linked to suppression of the PI3K/Akt signalling pathway (Lin and Rao, 2023). In sepsis models, poly (lactide-co-glycolic acid)-encapsulated FMN (PLGA-FMN) significantly upregulates PRDM16 expression and activates the NRF2/GPX4 pathway, effectively inhibiting ferroptosis and reducing sepsis-associated multiorgan injury, including lung damage (Zheng et al., 2024).

*In vitro* mechanistic studies have further elucidated the intricate mechanisms of action of FMN. FMN exerts anti-inflammatory effects on RAW264.7 mouse macrophages by upregulating SIRT1 in a PPARδ-dependent manner, which in turn suppresses the release of HMGB1, a late-phase inflammatory mediator (Li, 2024). FMN may affect NF-κB activity through direct suppression and through SIRT1-mediated upstream deacetylation, reducing p65 nuclear translocation and inflammatory gene expression. It also promotes Nrf2 nuclear translocation and target gene expression (e.g., HO-1 and NQO1) by decreasing reactive oxygen species (ROS) levels and NOX activity, disrupting the interaction between Nrf2 and Keap1, and preventing Nrf2 degradation. This creates an antioxidant–anti-inflammatory feedback loop. The ability of FMN to inhibit NLRP3-mediated pyroptosis and NRF2/GPX4-dependent ferroptosis highlights its role in targeting key cell death pathways in ALI, making it a regulator of critical “hub pathways” in ALI (Zheng et al., 2024). Additionally, FMN effectively reduces the expression of inflammatory factors such as TNF-α, IL-1β, and IL-6 by inhibiting the expression of inflammation-related factors such as IL-33, ST2L, and TRPA1, thus ameliorating lung inflammatory responses (Shi et al., 2024).

**FIGURE 1:**
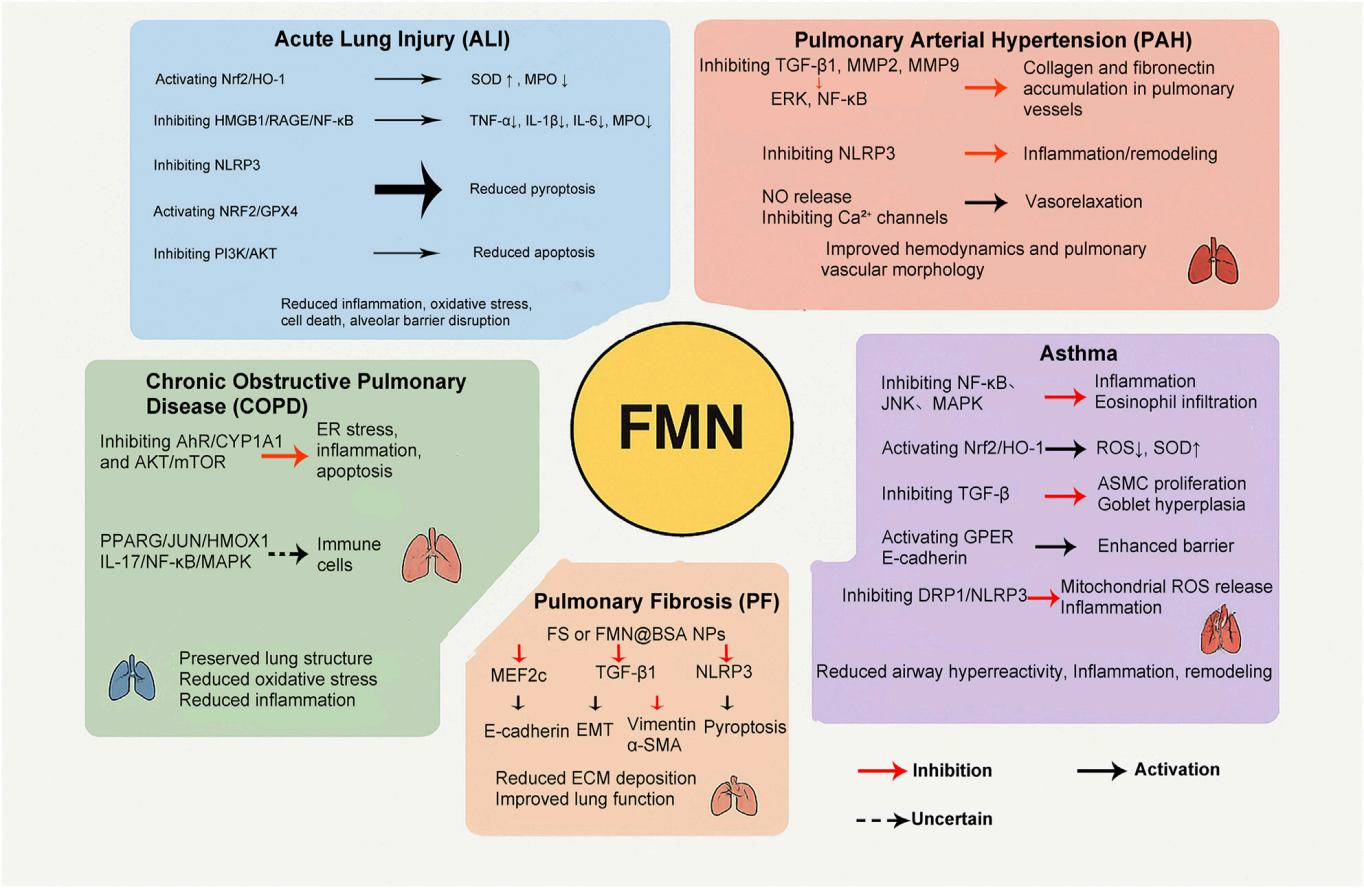
FMN core signaling axes in lung disease.

A critical synthesis of these findings reveals that the protective effect of FMN against ALI is multifaceted, as it targets both oxidative stress (via Nrf2/HO-1) and inflammation (via NF-κB and PI3K/Akt) almost concurrently. While Nrf2 activation is a consistent theme, upstream inflammatory inhibition (e.g., NF-κB) might be equally crucial for mitigating the initial insult. The relative contribution of each pathway may depend on the aetiology of ALI (e.g., sepsis vs. hyperoxia). Future studies should prioritize elucidating the precise molecular interactions between FMN and these signalling hubs and exploring potential crosstalk between these pathways (Figure 1).

## 3 FMN alleviates PAH by inhibiting vascular remodelling, inflammation, and calcium dysregulation

PAH is a progresive and deadly condition characterized by high pulmonary arterial pressure, vascular remodelling, and right heart failure. Existing treatments are not very effective, highlighting the need for new therapeutic strategies. FMN, known for its anti-inflammatory, antioxidant, and vasorelaxant effects, has potential as a treatment for PAH.

A consistent theme across *in vivo* studies is the strong ability of FMN to inhibit pulmonary vascular remodelling, a hallmark of PAH progression. FMN treatment clearly improves haemodynamics and pulmonary vascular structure and blocks vascular remodelling caused by monocrotaline (MCT) (Cai et al., 2019; Wu et al., 2020; Xudong et al., 2019). The primary mechanism involves inflammation–remodelling coupling. FMN reduces the levels of remodelling-related molecules such as TGF-β1, MMP-2, and MMP-9, which in turn decreases the activation of the ERK and NF-κB pathways (Cai et al., 2019; Wu et al., 2020). Specifically, it inhibits TGF-β1 signalling, leading to decreased ERK and p65 phosphorylation, restricted NF-κB nuclear translocation, and reduced proinflammatory gene activity. It has anti-inflammatory effects because it decreases collagen and fibronectin accumulation in pulmonary vessels, directly slowing PAH progression (Wu et al., 2020; Xudong et al., 2019). FMN also reduces inflammatory responses because it inhibits the NLRP3 signallingpathway, which helps reduce pulmonary arteriole remodelling (Wang et al., 2019). These actions are important because FMN downregulates the expression of MMP-2, which is typically upregulated in pulmonary artery endothelial cells (PAECs) during hypoxia-induced PH and is linked to disease severity and mortality, emphasizing the key regulatory role of FMN in the remodelling–inflammation–ECM triad (Arvidsson et al., 2022; Liu et al., 2018).

In addition to its anti-inflammatory and anti-remodelling effects, FMN has significant vasorelaxant and calcium (Ca^2+^) homeostatic properties. FMN promotes vasorelaxation both in an endothelium-dependent manner, which involves the release of nitric oxide (NO), and through endothelium-independent mechanisms, which are linked to the blockage of voltage-dependent Ca^2+^ channels and the release of intracellular Ca^2+^ (Sun et al., 2011). In hypertensive rat models, FMN gradually reduces arterial pressure, demonstrating its potential as an antihypertensive agent (Sun et al., 2011). These findings demonstrate that FMN can mitigate PAH by addressing vascular remodelling, inflammation, and haemodynamic issues simultaneously.

Although promising results have been obtained in animal models, research on the cellular mechanisms of FMN in PAH is limited, and robust clinical data on its safety and pharmacological efficacy in humans are lacking. Additionally, comprehensive long-term pharmacokinetic and pharmacodynamic profiles are insufficient. Future studies should delve deeper into cellular and molecular mechanisms, and high-quality clinical trials are needed to confirm the safety and pharmacological efficacy of FMN, possibly through exploration of its potential in combination therapies (Figure 1).

## 4 FMN mitigates COPD progression by reducing oxidative stress, ER stress, and epithelial cell apoptosis

COPD is a serious lung condition characterized by long-term airflow restriction due to chronic inflammation and airway changes (Barat et al., 2025). Current treatments often do not halt disease progression. FMN, an isoflavone with anti-inflammatory and antioxidant benefits, is a promising treatment option for COPD.

Preclinical studies have shown that FMN protects against cigarette smoke-induced COPD, a major global health issue. In mice, FMN reduces inflammation, oxidative and ER stress, apoptosis, and lung damage (Li et al., 2024). Its benefits in COPD are linked to the modulation of key intracellular pathways, particularly the suppression of endoplasmic reticulum (ER) stress and apoptosis, distinct from its broader anti-inflammatory effects in ALI and vascular remodelling in PAH.

*In vitro* experiments have revealed that FMN treatment inhibits the activity of the AhR/CYP1A1 and AKT/mTOR pathways, both of which are activated by smoke toxins and lead to oxidative stress, inflammation, and cell survival issues, in BEAS-2B cells exposed to cigarette smoke extract (Li et al., 2024). By blocking these pathways, FMN preserves epithelial cell integrity and mitigates smoke-induced damage, which is crucial for preventing COPD.

FMN, found in traditional Chinese medicines such as Qibaipingfei capsules and Bufei decoction, helps regulate immune responses in COPD. Network pharmacology suggests that FMN affects immune cells such as macrophages and T cells by modulating genes such as PPARG, JUN, and HMOX1 and pathways such as those involving IL-17, NF-κB, and MAPK (Wang et al., 2022; Jiao and Zhang, 2023). Although this the mechanisms of action of FMN are beyond the main focus of the present article, this study provides valuable insights.

FMN can treat COPD treatment by targeting ER stress, epithelial cell apoptosis, and the interaction of inflammatory and oxidative pathways. Its ability to protect bronchial epithelial integrity and mitigate ER stress-induced damage in COPD is distinct from its broader anti-inflammatory roles in ALI and its vascular effects in PAH (Figure 1).

## 5 FMN improves asthma outcomes by suppressing inflammation, enhancing antioxidant defences, and reducing airway remodelling

Asthma is characterized by long-term airway inflammation that causes wheezing, breathlessness, chest tightness, and coughing (Pradhan et al., 2025). Despite treatment, many patients continue to suffer symptoms and airway damage. FMN, known for its anti-inflammatory and antioxidant effects, shows promise in relieving asthma symptoms and underlying issues.

*In vivo* experiments have provided compelling evidence for the pharmacological efficacy of FMN in allergic asthma models. In mice with ovalbumin (OVA)-induced asthma, FMN treatment significantly improves lung function and alleviates pulmonary inflammation (Yi et al., 2020), as indicated by a reduction in the infiltration of inflammatory cells, specifically eosinophils, and decreased levels of key Th2 cytokines (IL-4, IL-5, and IL-13) and chemokines (CCL5/RANTES and CCL11/eotaxin-1), as well as IgE (Yi et al., 2020; Chen et al., 2024). Furthermore, FMN markedly reduces goblet cell hyperplasia and collagen deposition, indicating its ability to mitigate airway remodelling. FMN also effectively reduces oxidative stress by lowering ROS levels and increasing SOD activity (Yi et al., 2020; Chen et al., 2024). FMN protects against asthma by modulating key signalling pathways, inhibiting inflammatory pathways such as the NF-κB and JNK pathway, reducing proinflammatory cytokine levels and immune cell activation, and activating the Nrf2/HO-1 antioxidant pathway to counter oxidative damage (Yi et al., 2020). This dual action is essential for alleviating asthma symptoms.

The impact of FMN extends to other aspects of allergic inflammation. It reduces allergic inflammation by preventing histamine release and lowering proinflammatory cytokine levels, thereby decreasing hypersensitivity reactions (Xu and An, 2017). FMN strengthens the epithelial barrier through the G protein-coupled oestrogen receptor (GPER), potentially reducing allergen penetration, inflammation, and airway hyperreactivity (Yuan et al., 2020; Li et al., 2018). Additionally, FMN reduces the production of cytokines, including thymic stromal lymphopoietin (TSLP), by controlling E-cadherin expression in bronchial epithelial cells, which is crucial for Th2-type inflammation and asthma (Li et al., 2018). In an immunosuppressed mouse model, FMN increases immune functions by stimulating CD3^+^ T and CD20^+^ B lymphocyte proliferation in the thymus and spleen and increasing serum IL-2 and IL-4 levels (Zhang et al., 2020).

*In vitro* studies have further illuminated the cellular mechanisms of action of FMN in asthma. FMN suppresses the growth and activation of airway smooth muscle cells (ASMCs) triggered by transforming growth factor-beta (TGF-β), a major factor in airway remodelling (Tay et al., 2019). This action is vital for preventing irreversible airway structural changes in chronic asthma. Additionally, FMN inhibits the mitogen-activated protein kinase (MAPK) pathway, reducing eotaxin-1 production by airway fibroblasts, which contributes to eosinophilic inflammation (Zhou et al., 2012). FMN also decreases allergen-induced ROS production in BEAS-2B cells, inhibits DRP1 overexpression, stabilizes the mitochondrial membrane potential, and reduces mitochondrial ROS release, thereby preventing NLRP3 inflammasome activation and inflammation (Chen et al., 2024).

FMN acts as a multitarget asthma treatment by inhibiting inflammatory signals, increasing the levels of antioxidants, restoring the epithelial barrier, and reducing airway remodelling. These actions help decrease airway hyperresponsiveness, inflammation, and structural changes in asthma. However, more research is needed to understand how FMN affects immune cell differentiation and its long-term effects on the disease (Figure 1).

## 6 FMN and its derivatives attenuate PF through antifibrotic mechanisms when delivered by advanced drug delivery systems

PF is a serious, often deadly condition characterized by excessive ECM buildup and tissue changes, resulting in reduced lung function. FMN has shown promise owing to its antifibrotic and anti-inflammatory effects, but its clinical use is limited by poor water solubility and low oral bioavailability. To overcome these issues, researchers are investigating structural changes and advanced delivery methods.

*In vivo* studies revealed that *formononetin*-7-sal (FS), an FMN derivative with improved solubility and bioavailability, significantly ameliorates bleomycin-induced pulmonary fibrosis in mice. FS decreases fibrosis scores, collagen deposition, and the expression of fibrotic markers such as hydroxyproline, vimentin, and α-SMA but increases E-cadherin levels by inhibiting the MEF2c signalling pathway (Zhao et al., 2018). Another study revealed that FMN@BSA nanoparticles show improved delivery to the lung, are enriched in lung tissue, block the NLRP3‒macrophage pyroptosis‒fibrosis axis, enhance lung function and extend survival in animal models (Ouyang et al., 2023). Other innovative strategies include FMN-PLGA-MSs, which, when delivered through intratracheal instillation, achieve sustained pulmonary release and effectively alleviate bleomycin-induced pulmonary fibrosis in mice, demonstrating that localized controlled delivery can overcome systemic limitations (Liu et al., 2025). These findings support the view that albumin nanoparticles and polymer-based inhalable carriers are promising future pulmonary drug delivery systems because of their biocompatibility, biodegradability, and capacity for targeted deposition in diseased lung regions (Kumbhar et al., 2025; Peng et al., 2024).

*In vitro* investigations have further corroborated these *in vivo* findings. FS inhibits TGF-β1-induced epithelial‒mesenchymal transition (EMT) and migration in A549 and L929 cells; downregulates vimentin, α-SMA, and Snail expression; and upregulates E-cadherin expression (Zhao et al., 2018). Similarly, FMN@BSA nanoparticles effectively inhibit NLRP3 inflammasome-driven pyroptosis in alveolar macrophages, disrupting the macrophage pyroptosis–fibrosis axis and reducing profibrotic signalling (Ouyang et al., 2023). These findings indicate that although the solubility and bioavailability of FMN are limited, structural modifications and nanodelivery systems greatly improve its delivery to the pulmonary system and enhance its pharmacological effects, suggesting that the use of optimal delivery systems can amplify the pharmacological efficacy of FMN in the treatment of pulmonary fibrosis (Figure 1).

## 7 Mechanistic studies of FMN across major pulmonary diseases highlight shared and distinct signalling pathways

**FIGURE 2:**
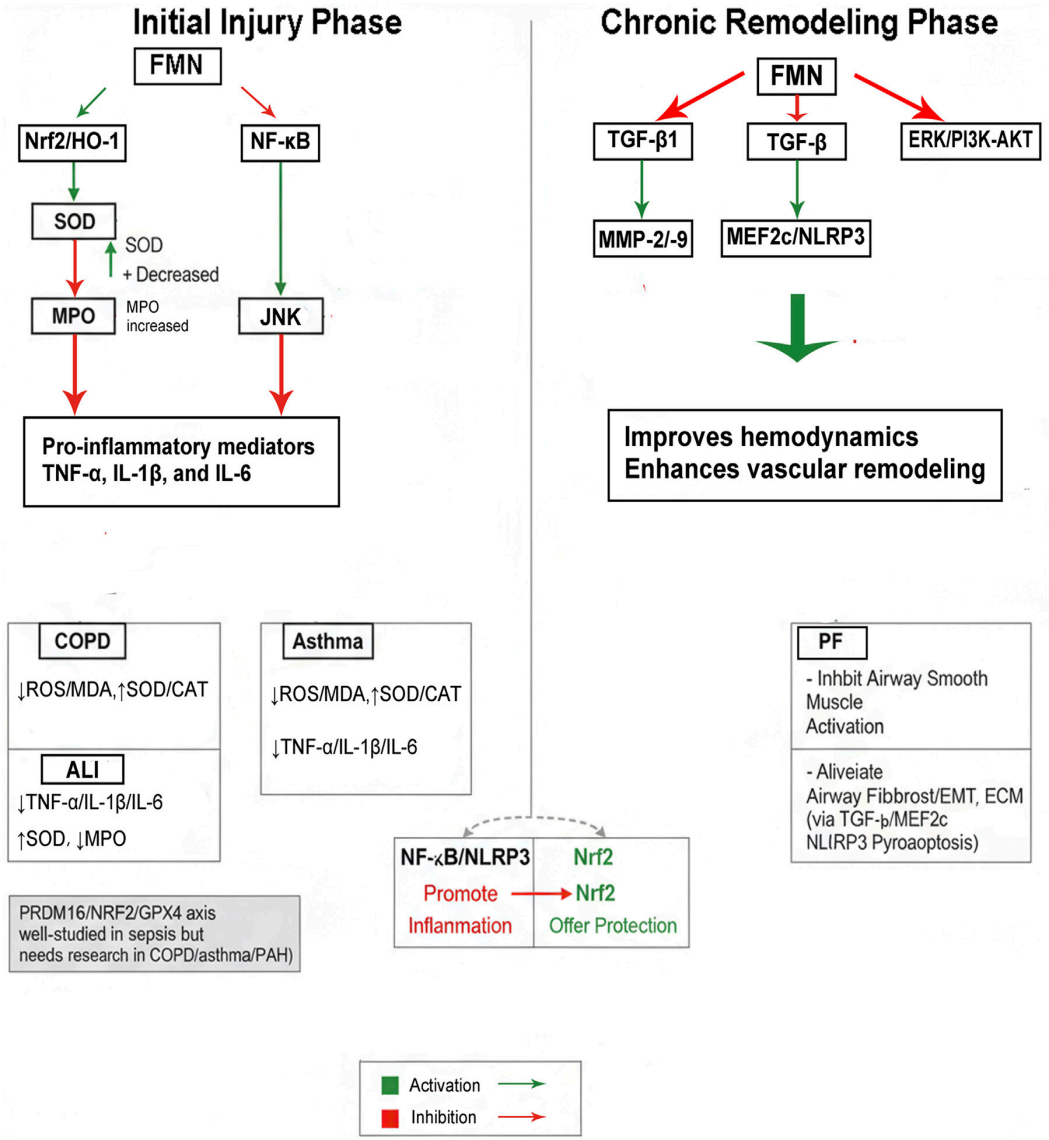
Mechanistic synthesis across pulmonary pathologies.

In ALI, COPD, asthma, PAH and PF, FMN consistently has dual effects: enhancing the antioxidant Nrf2/HO-1 pathway and suppressing NF-κB–mediated cytokine activity. In ALI models, FMN increases SOD levels, decreases MPO activity, and reduces the production of proinflammatory mediators such as TNF-α, IL-1β, and IL-6 by activating Nrf2/HO-1 signalling (Zhao et al., 2017; Chen et al., 2021; Ma et al., 2013; Li, 2024). Similar redox and inflammatory balance is observed in the affected epithelium of cigarette smokers with COPD and in asthma triggered by allergens, where FMN reduces ROS/MDA levels, restores SOD/CAT levels, and inhibits NF-κB/JNK signalling (Yi et al., 2020; Li et al., 2024; Derangula et al., 2021). Collectively, during the initial phase of injury marked by oxidative stress, the activation of Nrf2 (combined with NF-κB inhibition) appears to constitute the most pivotal regulatory mechanism (Figure 2).

In contrast, tissue remodelling and chronic progression are more closely related to the TGF-β signalling pathway. In PAH, FMN improves haemodynamics, enhances vascular remodelling, and reduces the activity of TGF-β1, MMP-2/9, and ERK/PI3K-AKT. In asthma, FMN inhibits TGF-β-induced airway smooth muscle activation (Cai et al., 2019; Sun et al., 2011). In PF, FMN derivatives alleviate fibroblast/epithelial‒mesenchymal transition and ECM deposition, indicating that TGF-β/MEF2c signalling and inflammasome-related pyroptosis are key mechanisms (Ouyang et al., 2023; Zhao et al., 2018). Thus, TGF-β signalling is crucial for structural remodelling and chronic conditions, with NF-κB/NLRP3 promoting inflammation and Nrf2 protecting cells (Figure 2).

Nonetheless, several important uncertainties remain, preventing definitive causal interpretation of these findings. First, mechanisms vary by cell type, with differences observed between macrophages, airway epithelial cells, and airway smooth muscle cells in COPD and asthma models. Second, the intricate interplay between pathways complicates the attribution of specific mechanisms, as ERK and PI3K–AKT can function either upstream of NF-κB or downstream of TGF-β, contingent upon the contextual framework. Consequently, temporal studies are crucial for establishing the hierarchical organization of these pathways. Third, while the role of the PRDM16/NRF2/GPX4 axis in sepsis-related injury has been well studied, more research is needed to understand its role in COPD, asthma, and PAH. Finally, although improved formulations have enhanced the pharmacological efficacy of FMN in rodents, human studies on the dose–response relationship and safety in PAH, COPD, asthma, and PF are insufficient.

## 8 Outlook

Our review highlights the promising therapeutic potential of FMN for various lung diseases, including ALI, PAH, COPD, asthma, and PF. Preclinical studies have shown that FMN exerts strong anti-inflammatory, antioxidant, and antifibrotic effects through pathways such as the Nrf2/HO-1, NF-κB, PI3K/Akt, ERK, and NLRP3 inflammasome pathways. The ability of FMN to target multiple disease mechanisms makes it a promising treatment for complex lung conditions.

Despite these encouraging findings, several critical areas require further investigation to translate the preclinical promise of FMN into clinical reality.

To further clarify the effects of FMN on the NF-κB, JNK, Nrf2/HO-1, and inflammasome pathways, spatial transcriptomics and single-cell multiomics studies are needed. Detailed research on mechanisms such as autophagic flux, apoptosis, ferroptosis, pyroptosis, mitochondrial dynamics, and interactions among the matrix, immune system, and epithelium is essential. These studies may help to identify predictive biomarkers, such as high NLRP3 expression or oxidative stress-related markers, to develop effective combination therapies for pulmonary fibrosis or identify bronchodilators/inhaled glucocorticoids that are effective for chronic obstructive pulmonary disease/asthma patients.

Optimized delivery systems: The inherent limitations of FMN, such as its poor water solubility and bioavailability, necessitate the continued development of advanced delivery strategies. Nanoparticle formulations (e.g., FMN@BSA nanoparticles and PLGA-encapsulated FMN) have shown superior pharmacological efficacy and targeted delivery. Future research should focus on optimizing these systems for specific lung diseases, potentially exploring inhaled delivery for direct lung targeting in asthma and COPD or sustained-release formulations for chronic conditions such as PAH and PF.

Toxicology and safety assessment: While FMN is generally considered safe, rigorous toxicology studies are needed to assess the potential side effects of FMN, particularly when it is administered over the long term or in vulnerable patient populations.

Clinical translation: The ultimate goal is clinical application. Achieving this goal requires well-designed, placebo-controlled clinical trials to evaluate the safety, tolerability, and efficacy of FMN in human patients with various pulmonary pathologies. Identifying appropriate biomarkers for patient selection and monitoring the treatment response will be crucial for successful translation.

FMN is a natural compound with significant potential to treat progressive lung diseases. To transform this promising preclinical agent into a safe and effective therapy for patients, we must accelerate our efforts; this involves gaining a deeper mechanistic understanding, developing innovative delivery systems, and conducting thorough clinical validation.
